# Depth- and temperature-specific fatty acid adaptations in ctenophores from extreme habitats

**DOI:** 10.1242/jeb.242800

**Published:** 2021-11-05

**Authors:** Jacob R. Winnikoff, Steven H. D. Haddock, Itay Budin

**Affiliations:** 1 Monterey Bay Aquarium Research Institute, 7700 Sandholdt Rd., Moss Landing, CA 95039, USA; 2 Department of Ecology and Evolutionary Biology, University of California Santa Cruz, 1156 High St., Santa Cruz, CA 95064, USA; 3 Department of Chemistry and Biochemistry, University of California San Diego, 9500 Gilman Dr., La Jolla, CA 92093, USA

**Keywords:** Biochemical adaptation, Ctenophores, Deep sea, Lipids, Pressure, Temperature

## Abstract

Animals are known to regulate the composition of their cell membranes to maintain key biophysical properties in response to changes in temperature. For deep-sea marine organisms, high hydrostatic pressure represents an additional, yet much more poorly understood, perturbant of cell membrane structure. Previous studies in fish and marine microbes have reported correlations with temperature and depth of membrane-fluidizing lipid components, such as polyunsaturated fatty acids. Because little has been done to isolate the separate effects of temperature and pressure on the lipid pool, it is still not understood whether these two environmental factors elicit independent or overlapping biochemical adaptive responses. Here, we use the taxonomic and habitat diversity of the phylum Ctenophora to test whether distinct low-temperature and high-pressure signatures can be detected in fatty acid profiles. We measured the fatty acid composition of 105 individual ctenophores, representing 21 species, from deep and shallow Arctic, temperate, and tropical sampling locales (sea surface temperature, −2° to 28°C). In tropical and temperate regions, remotely operated submersibles (ROVs) enabled sampling down to 4000 m. We found that among specimens with body temperatures 7.5°C or colder, depth predicted fatty acid unsaturation levels. In contrast, in the upper 200 m of the water column, temperature predicted fatty acid chain lengths. Taken together, our findings suggest that lipid metabolism may be specialized with respect to multiple physical variables in diverse marine environments. Largely distinct modes of adaptation to depth and cold imply that polar marine invertebrates may not find a ready refugium from climate change in the deep.

## Abstract (precis)

**Summary:** Global sampling of comb jellies reveals distinct fatty acid compositions in cold versus deep habitats, suggesting that high hydrostatic pressure requires specialized adaptation of lipid membranes.

## INTRODUCTION

In the deep ocean, life functions under a set of conditions totally foreign to humans: the temperature is near freezing and hydrostatic pressure reaches up to 1100 times that at sea level. Deep-living organisms are known to tolerate these conditions through differences in membrane lipid composition (Shillito et al., 2020), ‘chemical chaperone’ content (Yancey et al., 2014), and temperature- and pressure-resilient features in protein structure (Dahlhoff and Somero, 1991; Morita, 2003; Gerringer et al., 2017; Lemaire et al., 2018). However, definitive biochemical signatures of adaptation to specific environmental parameters remain elusive. Identifying distinct signatures has twofold utility: fundamentally, their existence implies selective forces endemic to near-freezing water and to the deep sea, e.g. that of hydrostatic pressure. Practically, the type and extent of adaptation required for survival in the deep informs whether and how species threatened by increasing sea surface temperature might use deep water as a refugium (Cottin et al., 2012).

The fluidity and phase of membrane lipids are acutely sensitive to both temperature and pressure (Somero, 1992; Hazel, 1995). Temperature effects on membranes have been extensively studied and exhibit a common pattern: for a lipid bilayer of fixed composition, cold temperature increases viscosity, while warm temperature increases fluidity. Changes in membrane permeability and intra-membrane diffusion of compounds such as ubiquinone accompany these perturbations (Budin et al., 2018). Extreme cold hardens the bilayer into a gel phase that limits diffusion within the membrane and promotes mechanical defects (Hazel, 1995; Shoemaker and Vanderlick, 2003), while extreme heat produces inverted lipidic phases, either hexagonal or cubic, in which the orientation of lipids is the reverse of that in a bilayer (Toombes et al., 2002). The temperature–fluidity relationship and phase-break thresholds are dependent on lipid composition and are tightly controlled in biological systems (Behan-Martin et al., 1993; Gershfeld et al., 1993; Logue et al., 2000). Mechanisms for regulating membrane homeostasis have drawn the attention of biologists for over half a century, in the course of which multiple adaptive strategies and selective mechanisms have been identified. One of the most common adaptive strategies involves increasing acyl chain unsaturation at low temperatures (Haest et al., 1969), which fluidizes the membrane and depresses its gel point. Shortening of saturated acyl chains was later observed as a parallel strategy in *E. coli* at strongly sub-optimal temperatures of 10–20°C (Suutari and Laakso, 1994). The mix of hydrophilic head groups incorporated into membrane phospholipids has also been found to change with environment and is thought to primarily affect the inverted phase transition (Hazel and Landrey, 1988). The homeoviscous adaptation hypothesis proposes that cells need to maintain membrane fluidity within a narrow range across temperatures (Sinensky, 1974; Hazel, 1995), driving lipidome adjustments. Alternatively, in homeophasic adaptation, the primary selective driver is a need to control gel and inverted phase transitions (Linden et al., 1973; Hazel, 1995). It has since become clear that both fluidity and phase are of paramount biological importance: fluidity controls cellular respiration (Budin et al., 2018) and ion permeability (Lande et al., 1995), while phase dictates the formation of lipid rafts (Simons and Vaz, 2004), the ability of membranes to fuse and bud (Siegel and Epand, 1997), and in extreme cases, whether a membrane forms at all. The relative importance of homeoviscous and homeophasic adaptation likely varies among organisms, membranes, and temperature conditions (Williams, 1998). Since homeoviscous adaptation is mainly dependent on phospholipid acyl chains, we have interpreted our fatty acid data in this context.

Effects of hydrostatic pressure on biological membranes have received much less study than effects of temperature. Within the native liquid-crystalline phase, the effect of high pressure resembles that of low temperature, and the two are essentially additive in promoting ordering, viscosity, and thickness of the bilayer (Macdonald, 1984). In membranes isolated from goldfish, the ordering effect of 1000 m seawater (100 bar) pressure is roughly equivalent to that of a 1.5°C drop in temperature (Chong et al., 1983). This relationship suggests that substantial acclimation or adaptation would be required for a deep-living organism or lineage to venture into even the coldest shallows, and vice versa. Nonetheless, some deep-sea species do emerge into surface waters at high latitude (Fosså, 1992). Whether predominantly polar species can accomplish the opposite feat in the face of climate change remains an open question. Insight into the mechanisms of polar emergence and viability of deep refugia requires data on the natural adaptation of animal membranes to high hydrostatic pressure, few of which have been gathered to date.

The metabolic pathways responsible for lipidomic adjustment have been identified at the biochemical level, but the regulatory networks in control of these pathways are a subject of ongoing study, especially in animals. The two signal transduction pathways that have been characterized, in bacteria and yeast, maintain homeoviscosity through transcriptional control of desaturase enzymes (Cybulski et al., 2010; Ballweg et al., 2020). Although their endogenous fluidity sensors are unknown, animals can acclimate similarly: in liposomes from the mussel *Mytilus californianus*, a fluidity increase was measured just 2.5 h after a temperature drop of 12.5°C (Williams and Somero, 1996). Animal lipid adaptation can also involve behavior: flies, for instance, have been found to actively alter their diet for greater intake of PUFAs during exposure to cold (Brankatschk et al., 2018).

Owing to the combination of parallel and distinct effects of pressure and cold on membranes, lipid composition presents a promising space in which chemical signatures of deep-sea adaptation can be identified. There are, however, challenges inherent to isolating temperature from pressure effects in the marine environment, foremost of which is the confounding decline in water temperature with depth in most parts of the ocean. Two sampling approaches are typically used to address this problem. The first approach is to investigate hydrothermal vent organisms, which are adapted to high pressure at temperatures comparable to those of tropical surface waters (e.g. Dahlhoff and Somero, 1991; Yancey, 2005), though physiological temperatures are difficult to estimate at vents because of the steep spatial gradients (Chevaldonné et al., 2000). Additional abiotic factors of the vent environment, such as sulfides, further complicate this approach (Grieshaber and Völkel, 1998). A second strategy is to sample from polar surface waters, where temperatures of −2 to 5°C fall within a typical range for the mesopelagic to abyssal zones (e.g. Cossins and Macdonald, 1989; Low and Somero, 1976). This permits comparison across a depth range of kilometers while holding temperature essentially constant, and captures greater diversity than sampling constrained to epipelagic and vent habitats. While it would be ideal to employ both sampling strategies, this has yet to be accomplished by any single study.

Even with the inclusion of polar samples, comparison of adaptive strategies across true oceanic extremes requires an interspecific approach. Some ectothermic species inhabit ranges spanning thousands of meters depth (Havermans et al., 2011) or tolerate tens of degrees Celsius variation in temperature (Dietz and Somero, 1992); however, we are unaware of any panmictic populations of adult animals encompassing epipelagic to abyssal depths or polar to tropical temperatures. Statistical regression methods have been developed that account for phylogenetic structure between samples, enabling inclusion of a broad diversity of species that are not necessarily environmental generalists (Martins and Hansen, 1997). Just as the strength of ordinary regressions can benefit from a balanced distribution of data points along the axes, phylogenetic regressions can derive statistical power from an appropriate balance of evolutionary relationships and phenotypes among samples. The most informative comparisons, in which phenotypic differences are more likely to be environmentally mediated, occur between closely related species living in different environments, and between distantly related species living in similar environments. Signals arising from other comparisons are more likely to be products of random genetic drift, and the residuals are down-weighted accordingly.

Ctenophores, also known as comb jellies, constitute an invertebrate phylum that is well suited to both phylogenetic regression and comparative study across marine environmental extremes. As gelatinous ectotherms, ctenophores have no mechanical nor thermal protection for their cells. Thermal and pressure-induced stresses must be borne directly by biomolecules. Therefore, all ctenophores are likely to exhibit biochemical signatures of adaptation to temperature and depth. A diverse array of ctenophores is present throughout the world ocean, living at −2°C to 30°C, and from the surface to over 7000 m depth (Lindsay and Miyake, 2007). Few invertebrate taxa are known to span comparable habitat diversity, and in many cases, the most extreme environments tend to host only one or two specialist lineages. In contrast, representatives of multiple ctenophore lineages, such as the mertensiids, lobates and platyctenes, are present in similar, often extreme, habitats, and some lineages (e.g. genus *Lampea*) have diversified to colonize disparate environments (Fig. S1), furnishing the informative interspecific comparisons described above. This evolutionary pattern may be attributable to long divergence times between extant ctenophores: while their last common ancestor has not been dated because of a lack of fossils, the common ancestor shared by ctenophores and other metazoa is several hundred million years old (Dunn et al., 2014), leaving abundant time for adaptive specialization.

In this study, we leveraged the unique intersection of biogeographic, evolutionary and physiological properties found in the phylum Ctenophora to perform a comparative analysis that considers natural adaptation to depth and temperature simultaneously. This analysis benefited from our access to sequence data sufficient to estimate robust relative genetic distances between ctenophore species. Fatty acid composition provided an ideal phenotypic readout because of its known importance to homeoviscous adaptation, ease of measurement, and relative stability, which enabled the use of samples from −80°C archives and remote collection locales.

## MATERIALS AND METHODS

### Specimen collection

Most ctenophores were collected between 2016 and 2019 using blue-water SCUBA techniques (0–25 m depth), MBARI remotely operated vehicles (ROVs) *Ventana*, *Doc Ricketts* and *MiniROV* (20–4000 m depth), and Bongo and Tucker trawls (100–1200 m). All Arctic samples were collected during June and July 2018. Samples were either snap-frozen whole in liquid nitrogen, or else protected against oxidation with approximately 0.01% v/v butylated hydroxytoluene (BHT, MP Biomedicals) and frozen at −20°C. Samples were brought back to the laboratory within 1 month and stored long-term at −80°C. Detailed metadata for each sample are available at github.com/octopode/cteno-lipids-2021: see Data Availability.

### Total lipid extraction and fatty acid analysis

Whole ctenophores were homogenized in a Dounce grinder on ice, then extracted using the method of Bligh and Dyer (1959) with about 0.01% v/v BHT. Aliquots of lipid extracts were resuspended in toluene and transesterified using 2.5% v/v sodium methoxide (Sigma) in dry methanol at 50°C for 30 min. Under these conditions, phospholipid acyl chains transesterify fully within 5 min and those from acylglyerols within 10 min. Wax esters transesterify more slowly, and free fatty acids do not react detectably (Christie, 1993). The resulting fatty acid methyl esters (FAMEs) were then extracted in hexane before analysis.

FAMEs were analyzed using gas chromatography-mass spectrometry (GC-MS). Samples were run on a 60 m DB25 column in an Agilent 8890 GC coupled to a 5977B mass analyzer. The GC was programmed to ramp from 40 to 230°C over 20 min, then hold for 6 min. FAMEs were identified and quantified using external standards: a 37-component standard mix (Supelco), and an equimass mixture of C18:4 (Cayman Chemical) and C22:5 (NuChek Prep) methyl esters. C20:1(*n*-9) and C22:1(*n*-9) fatty alcohol standards (NuChek Prep) were also injected externally as an equimass mixture. All standard mixes were analyzed at eight different split ratios. The slope of the integral vs split curve for each standard compound was used to determine its mass ionization coefficient, which was subsequently divided by its molar mass to obtain a molar ionization coefficient. These coefficients were then used to calculate mole fractions of each known compound in each sample (Table S1, Fig. S2). Mole fractions were used to calculate the double bond index (DBI) and mean chain length for each sample as in Vornanen et al. (1999).

The identities of target compounds, as well as of BHT preservative and its oxidation products, were initially checked against the NIST17 mass spectral library and NIST MS Search software (https://chemdata.nist.gov) and confirmed using the external standards. Raw Agilent data files were converted using the Agilent GCMS Translator utility and analyzed using the relative quantitation workflow provided with our purpose-built tidychrom package (github.com/octopode/tidychrom) in the R environment (https://www.r-project.org/). Single-ion integration was performed on the base peak, except for coeluting compounds, which were integrated on the most intense ion tenfold more abundant than in the coeluting spectrum (separate_signals function with thres_ortho=0.9).

### Environmental data

Collection coordinates for all specimens were recorded by GPS to a resolution of 1 km or finer. For specimens collected by ROV, the depth and temperature were recorded at time of collection using the vehicle's main CTD package (SBE 19plusV2). For trawled specimens, these parameters were estimated using data from the nearest ROV dive or hydrocast occurring immediately before or after the trawl (station and dive numbers are available at github.com/octopode/cteno-lipids-2021; see Data Availability). All hydrocasts were conducted from R/V *Sikuliaq* using an SBE 911plus. For specimens collected on SCUBA, depth and temperature were recorded using a dive computer.

### Phylogenetically generalized regression analyses

**Fig. 1.**
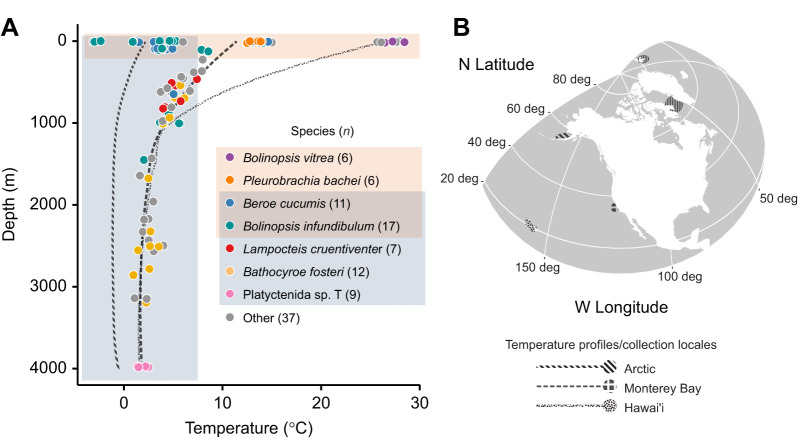
**Sampling through the water column and across latitude enables orthogonal analyses of depth and temperature adaptation in ctenophores.** (A) Depth and temperature of collection for each of the animals from which total lipids were extracted, total *N*=105. Of the 21 species collected, 8 with *n*≥6 are color-coded according to the species key. Two overlapping subsets of the data were used in different analyses: the pink region contains those used for temperature and oxygen saturation correlations, while the blue region contains those used in depth correlations. (B) Map of the collection locales, each of which is demarcated with a pattern matching a representative depth-temperature profile in A.

Linear relationships between environmental variables and lipidomic parameters were fitted using phylogenetic regressions (Grafen, 1989; Felsenstein, 1985). Briefly, this method estimates expected covariance in cross-species data: when there is large variation in residuals among closely related samples, those samples exert a stronger effect on the regression. The more distantly related the samples in question are, the weaker their effect becomes, based on the notion that the less ancestry they share, the more likely it is that their phenotypes drifted apart by chance (Symonds and Blomberg, 2014). Relatedness is derived from divergence times in a previously inferred phylogeny, and the function linking divergence time with expected residual covariance takes the form of a model of trait evolution through time. Some of these models allow for zero phylogenetic signal (i.e. an ordinary regression) as a special case (Pagel, 1997) We carried out phylogenetic regressions using the R package nlme (https://CRAN.R-project.org/package=nlme) under an Ornstein–Uhlenbeck model of trait evolution implemented in the corMartins function (Martins and Hansen, 1997) in the R package ape (https://CRAN.R-project.org/package=ape). The provisional ctenophore phylogeny used (reflected in Fig. S1 and available at github.com/octopode/cteno-lipids-2021) was generated by running OrthoFinder v.2.3.1 (Emms and Kelly, 2015) to completion with default parameters on 21 ctenophore transcriptomes sequenced from MBARI samples (Table S2) and assembled in-house with Trinity (Grabherr et al., 2011). The topology obtained was consistent with Townsend et al. (2020). Individuals were added to the tree as terminal polytomies before computing covariances. To limit covariation of environmental variables, regressions against temperature were constrained to specimens collected shallower than 200 m, and those against depth were limited to specimens obtained at temperatures colder than 7.5°C (orange area, Fig. 1A). These limits were chosen prior to performing regression analyses. For all regressions, familywise type I error rate was controlled across dependent variables by the method of Holm (1979).

## RESULTS

### Collections

Multiple collection methods were used to obtain ctenophores from the most diverse set of habitats possible. SCUBA, ROV and trawl sampling yielded 105 usable specimens (Fig. 1A). Forty-five individuals across 7 species were collected shallower than 200 m (orange area, Fig. 1A) and were included in the temperature analysis. Seventy-five individuals across 16 species were collected in water colder than 7.5°C, and thus included in depth analyses (gray area, Fig. 1A). The overlap of these slices contained 17 individuals across 3 species, which were included in all environmental correlations. Three individuals of 3 different species collected outside of either slice were omitted from environmental correlations but included in summary statistics and intercorrelations. Large amounts of BHT oxidation products were found in some samples, occasionally saturating the MS detector. These oxidation products co-occurred in samples with high fractions of polyunsaturated fatty acids (PUFAs), anecdotally suggesting that BHT was effective as an antioxidant for storage and transport.

### High-level trends

**Fig. 2.**
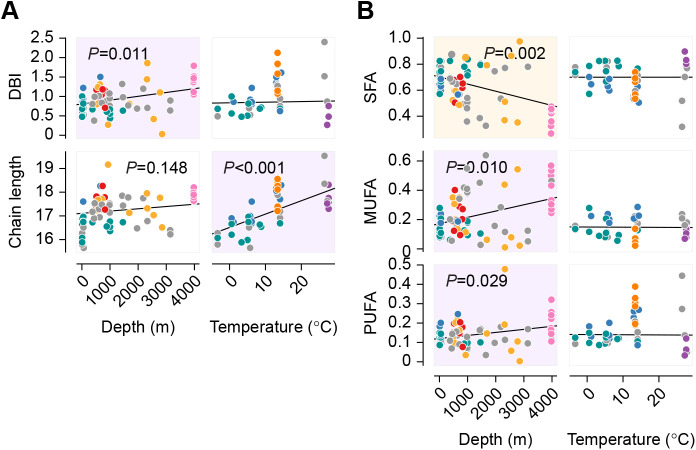
**Functionally important properties of the fatty acid pool respond distinctly to pressure and temperature.** Adjustments in DBI and chain length of phospholipid acyl groups are documented mechanisms of homeoviscous adaptation. (A) Phylogenetic regressions of both these summary variables against depth and temperature reveal distinct environmental responses: DBI increased significantly with depth, while mean chain length increased most strongly with temperature. (B) Phylogenetic regressions for fatty acid classes implicated in these responses: with increasing depth, SFAs are replaced to a significant degree by both mono- and polyunsaturated species. Consistent with temperature-independence of double bond count, none of the saturation classes varied with temperature. Depth analyses were constrained to temperatures ≤7.5°C, and temperature analyses to depths ≤200 m. Species are color-coded as in Fig. 1, *P* values <0.15 after Holm multiple testing corrections are displayed, and relationships found significant to alpha=0.05 are emphasized with a shaded background. Magenta denotes a positive and orange a negative correlation.

Owing to the complexity of animal fatty acid profiles, we first assessed summary properties of the even-chain fatty acid pool that have previously been implicated in environmental adaptation. Double bond index (DBI) and chain length of membrane lipids are important determinants of membrane fluidity (Ernst et al., 2016) and so were calculated as means weighted by mole fraction (Vornanen et al., 1999) (Fig. 2A). We found a significantly positive relationship between depth and DBI (Holm-adjusted *P*=0.011), as well as between temperature and chain length (*P*<0.001). To obtain a more detailed picture of fatty acid unsaturation, we calculated the total mole fraction of each fatty acid saturation class in each sample (Fig. 2B). The decline in saturated fatty acid (SFA) content with depth (*P*<0.002) was accompanied by increases in both monounsaturated fatty acid (MUFA) and PUFA fractions, with the effect size on MUFA being larger and marginally more significant (*P*=0.010 versus *P*=0.029). When ordinary least-squares regressions were performed for each species individually, and type I error controlled across dependent variables within the species, significant correlations were observed in *Beroe cucumis* and *Bolinopsis vitrea*. In *B. cucumis*, chain length increased and SFA decreased with temperature (*P*=0.007 and 0.039, *n*=10). In *B. vitrea*, SFA also decreased with temperature (*P*=0.041, *n*=6).

### Composition of fatty acid methyl esters and fatty alcohols

**Fig. 3.**
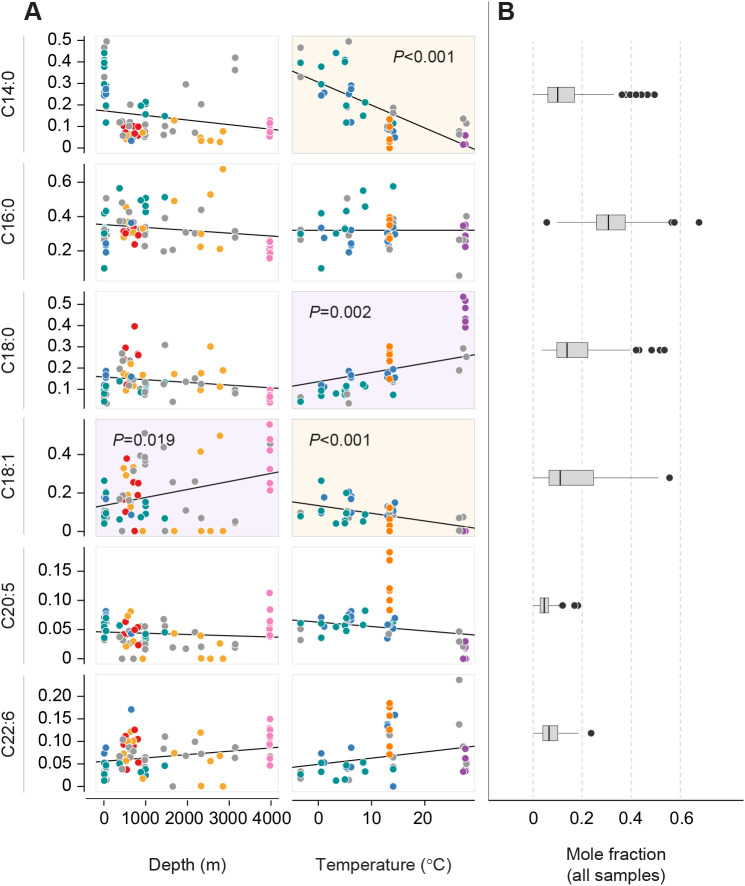
**Specific fatty acids drive phylum-wide trends in unsaturation and chain length.** Broad trends in acyl chain structure with depth and temperature are caused by variation in a subset of the 6 major fatty acids in ctenophores. (A) Phylogenetic regressions of the 6 predominant fatty acids against depth and temperature at the point of collection. Species are color-coded as in Fig. 1, with magenta- or orange-shaded plot backgrounds denoting significant positive or negative correlation, respectively. Increased temperature is associated with a significant exchange of C14:0 for C18:0, as well as a decrease in C18:1, while depth is associated with a significant increase in C18:1. (B) Distributions of the top 6 fatty acid methyl ester mole fractions in all samples, sorted by chain length and number of double bonds. Box plots are marked at the median and hinged at the first and third quartiles, with outliers >1.5 IQR from the median plotted individually.

To ascertain the metabolic pathways associated with lipid adaptation to the environment, we examined relative molar abundances of individual fatty acid methyl esters (FAMEs). Twenty-nine different FAMEs were detected at statistically significant levels across all ctenophores (one-tailed Student's *t*-test with Holm correction following the removal of six-sigma outliers; see Fig. S2). Of these, only 6 had mean mole fractions greater than 2.5%: C14:0, C16:0, C18:0, C18:1, C20:5 and C22:6. Proportions of these six major FAMEs are shown in Fig. 3B. Three of these six were significantly correlated with environmental parameters (Fig. 3A): C18:1 increased with depth (*P*=0.019); this was complemented by a significant decrease in total fraction of the top three SFAs (Fig. 2B). Of these, C14:0 displayed the steepest depth-related decline, but none of the individual trends were significant. Significant temperature trends among the major fatty acids described an exchange of C18:0 (*P*=0.002) for C14:0 (*P*≪0.001), and to a lesser degree for C18:1 (*P*<0.001) with decreasing temperature. Because fatty acid elongation and beta-oxidation both occur in two-carbon increments, C16:0 is a metabolic intermediate in this exchange, and its fraction was held fairly constant (around 0.35) across all environmental conditions. There was also a significant decrease in C18:1 with increasing temperature (*P*<0.001).

Despite their occurrence at low mole fractions, we tested environmental trends in all odd-chain fatty acids (OCFAs), since these are generally regarded as microbial metabolites and could shed light on ctenophore–microbial interactions. Six odd-chain FAMEs were consistently detected in ctenophores (Fig. S2), among which C15:0, C17:0 and C17:1 were most abundant, with mean mole fractions of 1.9, 2.1 and 0.75% (Table S1). C17:0 increased significantly with temperature up to a mole fraction of 8.0% (*P*<0.001), concomitant with a decrease in C17:1 (*P*=0.001), which is consistent with homeoviscous adaptation. Consistently high total OCFA fractions with means of 16.8 and 8.5% were observed in the species *Lampea* sp. and *Bolinopsis vitrea* (Table S1).

In addition to FAMEs, two monounsaturated long-chain fatty alcohols were detected in a subset of samples: C22:1(*n*-9) alcohol at mole fractions up to 4.6%, and trace amounts of C20:1(*n*-9) alcohol (up to 0.09%). These fatty alcohols were most likely liberated *in vivo* or during transesterification from wax esters used as energy storage compounds by ctenophores’ prey and ctenophores themselves (Graeve et al., 2008). Although the biological significance of fatty alcohols in ctenophores is not yet fully understood, they are ubiquitous in specimens from the Arctic and Antarctic circles (Phleger et al., 1998). Our most alcohol-rich samples also came from high latitudes, and environmental trends reflected this: total fatty alcohol fraction increased significantly with low temperatures (*P*<<0.001) encountered in polar surface waters and declined with depth (*P*=0.009).

## DISCUSSION

Our data demonstrate that ctenophores adjust largely distinct, biophysically relevant aspects of their lipidomes in response to depth and temperature. While more extensive sampling might demonstrate this ability at an individual, acclimatory scale in some species, we observed the most pronounced environment-composition trends across multiple species, suggesting that fatty acid composition is determined by both environmental and genetic components. The observation of general acyl chain adaptation to the environment concurs with decades of prior work on marine ectotherms, while that of distinct responses to depth (pressure) and temperature is novel, likely because our comparative lipidomic analysis is one of few (Pond et al., 2014; Taghon, 1988) to survey these two factors simultaneously. Mean chain length varied only with temperature among shallow samples, driven by a tradeoff between C14 and C18 saturated fatty acid content, while unsaturation varied predominantly with depth among cold-water samples, driven by a shift in the balance between the total SFA pool and monounsaturated C18:1. We will first consider features of diet and lipid metabolism, the proximate drivers of fatty acid composition, consistent with our dataset, and will subsequently discuss possible biophysical explanations, i.e. ultimate causes, for the patterns observed.

Diet and metabolism are proximally responsible for variation in animals’ fatty acid composition. Some of the compositional trends observed involved robust trophic markers and were thus attributable to diet. Other trends could have implicated both diet and metabolism and yet others were likely driven by metabolic pathways within ctenophores. The most striking diet-mediated patterns occurred in odd-chain fatty acids (OCFAs), which contain an odd number of carbon atoms. OCFA synthesis pathways have not been found in marine animals, but are widespread among bacteria, making these compounds *de facto* indicators of bacterial biomass in the food chain (Dalsgaard et al., 2003). We observed consistently high OCFA fractions in *Lampea* sp. (15.6-18.3%, *n*=4) and *Bolinopsis vitrea* (5.9-8.0%, *n*=6) (Table S1, Fig. S3A). This likely reflects dietary habit, as *Lampea* spp. specialize on salps, which consume bacteria-laden particles (Haddock, 2007). We also measured unexpectedly high fractions of C15:0 in one *Lampocteis cruentiventer* and two *Bathocyroe fosteri* specimens (16.4-22.4%), suggesting that these species occasionally ingest detritivores or sinking detritus. These observations represent an early step toward identifying OCFA vectors and sources in pelagic ecosystems. We detected one robust environmental pattern among OCFAs: an exchange of C17:1 for C17:0 with increasing temperature (Fig. S3A). Given the low levels observed in most species (Fig. S3B), it is unlikely that odd-chain fatty acids contribute ubiquitously to the environmental adaptation of ctenophores themselves, but this trend could reflect homeoviscous adaptation of microbial lipidomes to temperature.

The long-chain fatty alcohols found in some samples also appear to be dietarily derived. There are no conclusive data on ctenophores’ ability to synthesize these compounds, but in the Arctic species *Mertensia ovum*, high fatty alcohol content coincides with high abundance and consumption of herbivorous calanoid copepods that produce and accumulate fatty alcohols in the form of wax esters (Graeve et al., 2008). Curiously, the same study found that free C22:1 alcohol persisted longer in *Mertensia* after feeding than any other wax ester or alcohol. Our data were consistent with this finding: though we could not directly distinguish free from esterified fatty alcohols, C22:1(*n*-9) was the most abundant alcohol by 50-fold, and the fatty acids to which it is typically esterified in calanoids, C16:1 and C18:4 (Graeve and Kattner, 1992), were at trace levels comparatively. Multiple explanations have been proposed for the persistence of this particular compound in ctenophores: it could be catabolized slowly owing to a chain-length preference in the oxidizing enzymes, or it could be actively retained for its high energy density (Albers et al., 1996; Graeve et al., 2008). Our results align with previously published data (Albers et al., 1996; Graeve and Kattner, 1992; Graeve et al., 2008) suggesting that the slow catabolism and primarily dietary origin of C22:1 alcohol make it an excellent trophic marker at the secondary consumer level.

The most notable pattern observed among PUFAs was a significant depth-related increase in total PUFA (*P*=0.029), but not in either of the major PUFAs individually. Most animals are not able to synthesize PUFAs from MUFAs, but express elongases and desaturases active toward C18 polyunsaturated species (Monroig and Kabeya, 2018). If this is true for ctenophores, it would imply that the total PUFA fraction is constrained by diet, and further that feeding behavior could be critical for environmental adaptation, as observed in some copepods (Pond et al., 2014). The predominance of C20:5 and C22:6 over C18 PUFAs by roughly an order of magnitude (Fig. S2) likely reflects their incorporation at the sn2 position of phospholipids (Antonny et al., 2015; Manni et al., 2018). The strong positive intercorrelation of C20:5 and C22:6 (*P*<<0.001) suggests that they might be functionally interchangeable, with no strong metabolic tendency toward one at the expense of the other. In light of this, it is possible that the PUFA profile of a given individual somewhat resembles that of its food, but it is unlikely to provide much quantitative or specific information about ctenophore diet because PUFAs can be interconverted by many taxa across a range of trophic levels.

**Fig. 4.**
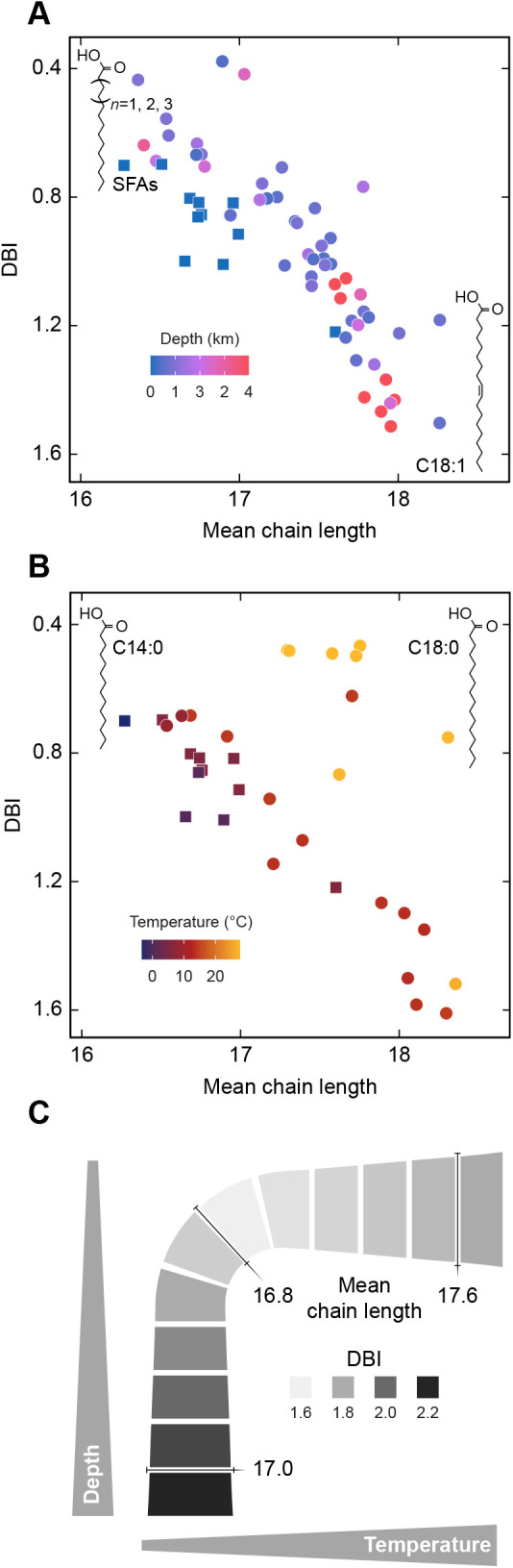
**Ctenophore acyl chain properties partition by habitat.** (A,B) To illustrate acyl chain properties characteristic of different habitats, individual ctenophores are plotted according to the DBI and chain length of their total fatty acids and colored using a continuous scale. The color scale in A indicates the depth of specimens collected colder than 7.5°C; that in B indicates temperature of those collected shallower than 200 m. Shallow and cold specimens present in both panels A and B are shown as square data points, and the structures drawn in the plot corners represent compounds driving the observations plotted nearby. (C) Summary of the linear models of double bond and chain length trends across sampled depth–temperature space, with axes analogous to those in Fig. 1A. Note the positive correlation between double bonds and chain length in A and B, owing to a longer-chain bias in ctenophore PUFAs. In A, shallow and deep animals cluster at opposite ends of this main diagonal trend, owing to an exchange of C14/16/18 SFA (shown in top left corner) for C18:1 (bottom right). In B, tropical shallow animals cluster to the right of the diagonal owing to a strong temperature effect on the ratio of C14:0 (top left) to C18:0 (top right).

Variation among C14:0, C16:0 and C18:0 SFAs was responsible in part for the observed chain length-temperature trend (Fig. 4B), and represents another chemical space in which both diet and metabolism might be at play. All three SFAs are present in common ctenophore prey (copepods) across a latitudinal gradient, however C18:0 is somewhat more abundant near the equator and C14:0 toward the poles (Kattner and Hagen, 2009). This alone might be sufficient to drive the pattern we observed in ctenophores, so further physiological study would be helpful in determining precisely how the SFA chain length difference is adaptive in ctenophores, their prey, or both. On the other hand, the enzymes elongation of long-chain fatty acids family member 6 (ELOVL6) and carnitine acyltransferase 1 (CAT1), which are rate-limiting for elongation and beta-oxidation of these fatty acids, are ubiquitous in animals (Castro et al., 2016), so ctenophores almost certainly could adjust their ratios metabolically provided they have the necessary regulatory pathways. Acclimation experiments under controlled diet could be used to determine whether active adjustment occurs in response to temperature.

Variation in the fraction of C18:1 MUFA, which increased in both deep and cold habitats, (Fig. 4A), is likely mediated by ctenophore metabolism. Robust, but sequentially smaller, intercorrelations occur between C18:1 and the C18, C16 and C14 SFAs (Fig. S4), consistent with active interconversion between C18:0 and C18:1 catalyzed by stearoyl-CoA desaturase (SCD1). This, and the strong correlations of C18:1 with both depth and temperature (*P=*0.019 and *P<*0.001, Fig. 3A), suggests that SCD1 could be an important enzyme for ctenophores when faced with excess dietary SFA or when moving to deeper or colder waters. Alternatively, the strong negative intercorrelations could be driven simply by the exclusive incorporation of C14-18 SFA and MUFA at the sn1 position in phospholipids (Antonny et al., 2015; Manni et al., 2018), with the SFA/MUFA ratio controlled by SFA catabolism (beta-oxidation by CAT1). This mode of adjustment would presumably occur when SFA and MUFA are both sufficiently abundant in the diet.

Irrespective of the proximate contributions of diet and metabolism, habitat depth and temperature appear to exert selective forces on ctenophore acyl chain composition, which can be viewed as ultimate causes for the compositional trends observed. Trends with both depth and temperature are readily explained by the homeoviscous principle: the ordering effects of high pressure and low temperature both appear to be compensated by biochemical adjustments known to promote membrane disorder. The basis for the difference in homeoviscous strategies visible in Fig. 2A is an intriguing subject requiring further study, as the acyl chain data are consistent with at least two biophysical explanations. One hypothesis is that membrane dimensions associated with fluidity could be differentially affected by the two variables: for instance, if low temperature increases viscosity primarily by thickening the bilayer, then shortened acyl chains might directly offset membrane thickness. Similarly, if pressure-induced ordering is caused mostly by a decrease in phospholipid spacing, then the kinked structure of unsaturated acyl chains could be employed to maintain this spacing. If there are such differences in the perturbation of bilayer structure caused by pressure and temperature, then different homeoviscous strategies might be required to maintain appropriate membrane dimensions and fluidity in shallow versus deep water.

A second hypothesis for different adaptive strategies is that homeophasic control has evolved in addition to homeoviscosity. The pressure–temperature equivalence values for transitions between the predominant liquid-crystalline phase and the less common gel and inverted phases are known to be different (So et al., 1993). Pressure protects against inverted phases more effectively than low temperature, and this could explain why shallow cold-adapted ctenophores maintain fluidity with acyl chain shortening instead of unsaturation (Fig. 2A): unsaturation facilitates inverted phases, whereas shortening raises the inverted phase transition temperature (Tenchov, 1991). Analogously, temperature tends to have a stronger effect on ordered phase transitions than pressure does, so chain shortening could also offset this by depressing the liquid-ordered or gel phase transition temperature (Cevc, 1991). Considering both these potential effects of chain length, chain shortening may effectively be a response to greater temperature variability in surface waters than in the deep.

Further work to test these biophysical hypotheses will require additional analytical approaches such as structural measurements of pressure and temperature effects on membrane dimensions and phase transitions of ctenophore-derived lipids (Gruner, 1985; Pabst et al., 2003). Biophysical data would be complemented by polar lipid and sterol composition profiles, since various membrane properties are known to depend on sterol content, phospholipid head group composition and the way acyl chains are paired under these headgroups (Cevc, 1991; Gruner, 1985). In particular, an enrichment of membrane-destabilizing headgroups such as phosphoethanolamine in deep-sea samples would suggest that the ability of membranes to invert (e.g. for vesicle budding and fusion) (Siegel and Epand, 1997) at high pressure is an important evolutionary selector. The combination of biophysical interrogation with more detailed lipidomic profiling will help elucidate the reason for alternative compensatory adaptations in cold and deep ctenophores.

In addition to biophysical constraints, a chemical driver in the form of oxidative stress could have explained alternative homeoviscous strategies. While phospholipids containing PUFAs are effective at promoting membrane fluidity (Brockman et al., 2007; Manni et al., 2018), they are also prone to damage by reactive oxygen species (ROS) of photochemical and mitochondrial origin (Hulbert et al., 2007; Xu et al., 2009). This might restrict the incorporation of PUFAs into membranes of ctenophores exposed to UV radiation and high ambient oxygen in sunlit, eutrophic surface waters and favor the shortening of saturated acyl chains as a fluidity maintenance strategy. It is notable that unlike similarly transparent-bodied cnidarians living at the same shallow depths, ctenophores lack any mycosporine-like amino acids for UV screening (Karentz et al., 1991) and might thus require a radical-tolerant lipidome. Though ctenophores are highly effective oxyregulators (Thuesen et al., 2005), mitochondrial ROS could also contribute to observed trends at interspecific and interpopulation scales: epipelagic ctenophore species tend to exhibit higher mass-specific respiration than their midwater relatives (Youngbluth et al., 1988), and some ctenophores respire up to 10 times faster when fed than when starved (Gyllenberg and Greve, 1979). Both photochemistry and cellular respiration could conceivably elevate ROS levels in ctenophores subjected to extremely high oxygen levels in the Arctic shallows during spring and summer (Eveleth et al., 2014). Although our study did not collect data sufficient to assess ROS constraints on lipidomes, this hypothesis could be tested in the future by sampling polar ctenophores along a seasonal gradient concurrently with optical profiles and CTDO data collection, and then assaying their tissue for superoxide dismutase activity and lipid peroxides alongside lipid analysis.

A potential limitation of this study is the use of total lipid extracts of whole ctenophores in FAME analysis. This approach was chosen mainly for practical reasons, since gelatinous animals become difficult to dissect after freezing. Chemical fractionation of over 100 samples would likewise have been cumbersome, since ctenophore total lipid content rarely exceeds 1% of wet mass (Nelson et al., 2000) and Bligh–Dyer extracts are initially very dilute because of the animals’ high water content. As a consequence, the composition data reported here offer no direct means to distinguish membrane fatty acids incorporated in phospholipids from those incorporated in triacylglycerides (TAGs) for energy storage. Fortunately, ctenophores appear to preferentially accumulate wax esters for energy storage when diet permits (Graeve et al., 2008), so TAG content rarely exceeds 5% of total lipid mass (Andrew et al., 1987; Nelson et al., 2000). Wax esters can be partially identified in GCMS data through their constituent fatty alcohols, while free fatty acids do not transesterify and are thus excluded from the data. In sum, the FAME compositions reported here should closely resemble the fatty acid ratios in combined membranes of each animal. As whole-tissue data, they are unlikely to reflect the makeup of any particular membrane (cytosolic, endoplasmic, mitochondrial, etc.), but are nonetheless useful for capturing adaptive responses that these structures may have in common.

Temperature and depth appear to shape the fatty acid composition of ctenophores in overlapping, yet distinct ways (Fig. 4C). The fundamental niches of ctenophores and perhaps other water-column organisms could thus be limited by both factors. Although our understanding of the precise biophysical and chemical mechanisms that set these limits will benefit from further study, it is clear that the physiological requirements for vertical range expansion can be multifaceted and include tolerance of high hydrostatic pressure. This dynamic underscores that many gelatinous organisms, although often portrayed as ecological beneficiaries of climate change (Morley et al., 2019), may not be capable of colonizing new parts of the water column on short timescales.

## Supplementary Material

Supplementary information
