# Supplementary material for: Depth- and temperature-specific fatty acid adaptations in ctenophores from extreme habitats
Source: J Exp Biol. 2021 Nov 5;224(21):jeb242800. doi: 10.1242/jeb.242800 (PMC8627573; doi:10.1242/jeb.242800)
Supplement: Supplementary information [file jexbio-224-242800-s1.pdf]

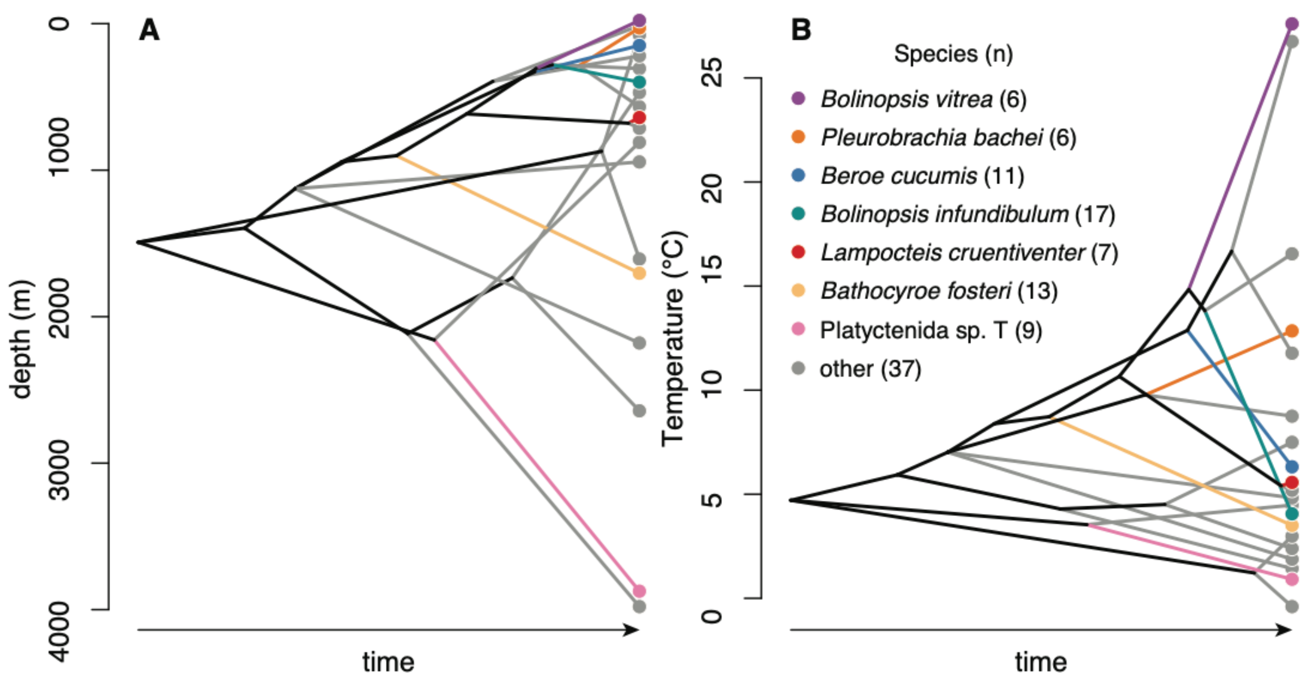

Fig. S1. Habitat convergence and divergence are ubiquitous in ctenophore evolution Brownian motion evolutionary reconstructions of ctenophore habitat (A) depth and (B) temperature both exhibit characteristics beneficial to phylogenetic regression. Habitat convergence is evident in the large number of intersecting lineages, while rapid divergence is indicated by recent nodes with their branches spread wide across the habitat axis. Note that this visualization does *not* imply that the last ctenophore ancestor lived circa 1500 m and 5°C; such an inference would be sensitive to rooting and taxon inclusion, and is outside the scope of this study.

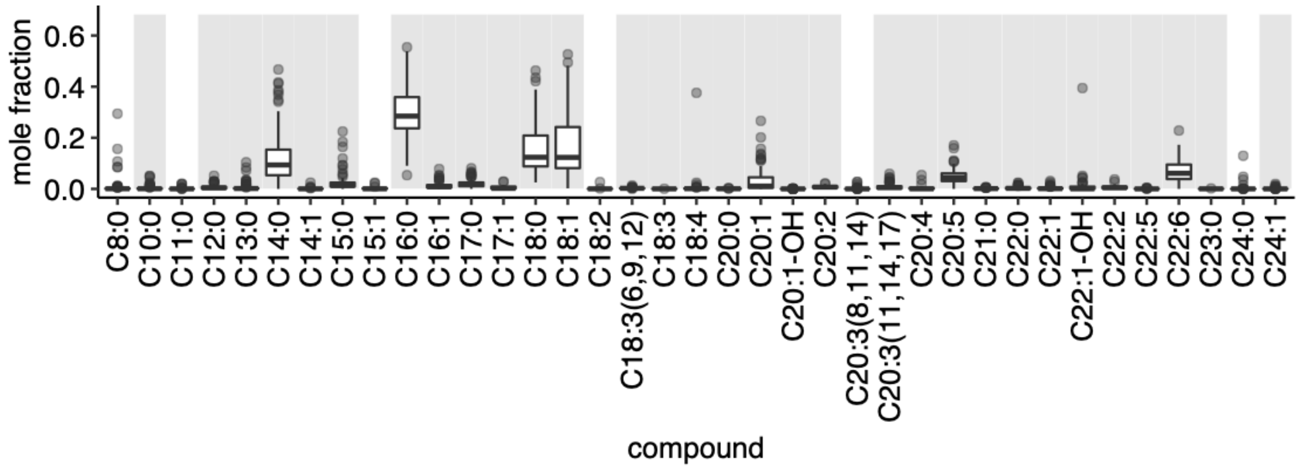

Fig. S2. Mole fraction distributions for all thirty-seven compounds quantified Compounds are sorted by increasing chain length and number of double bonds. Grey shaded columns denote compounds with mole fractions significantly different from zero across all 105 samples after Holm correction. Note that lack of shading does not invalidate values in that column; rather it indicates that the compound was not consistently detected in ctenophores.

Journal of Experimental Biology • Supplementary information

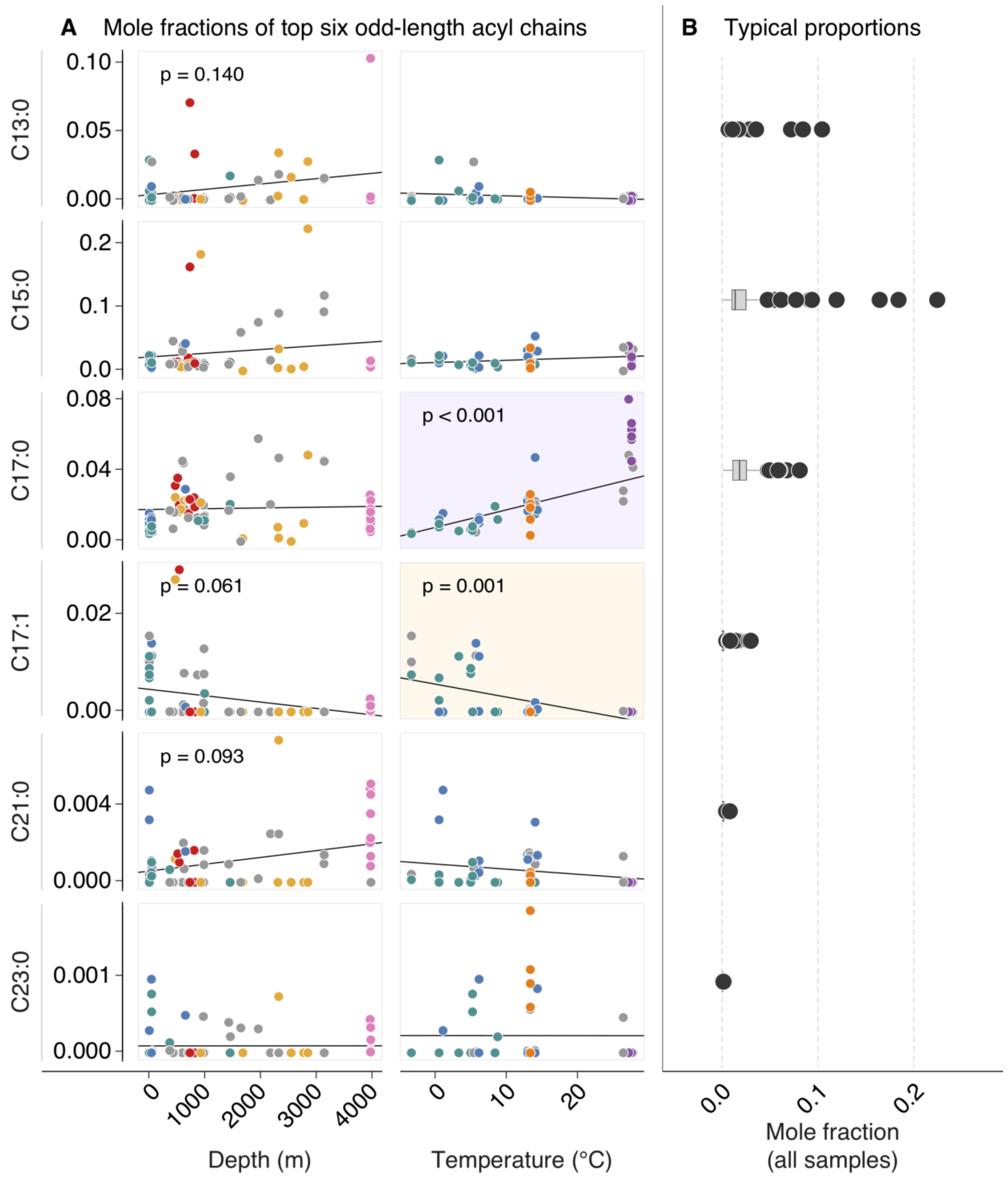

Fig. S3. Odd-chain fatty acids are characteristics of some species and habitats
(A) shows distributions of the relative abundances of six odd-chain fatty acid moieties that were consistently detected. (B) Phylogenetic regressions of each of these compounds against temperature and depth at the point of collection. Note the temperature trends in saturated and monounsaturated C17 fatty acids. Species are color-coded as in figures 2 and 3, with magenta- or orange-shaded plot backgrounds denoting significant positive or negative correlation.

Journal of Experimental Biology • Supplementary information

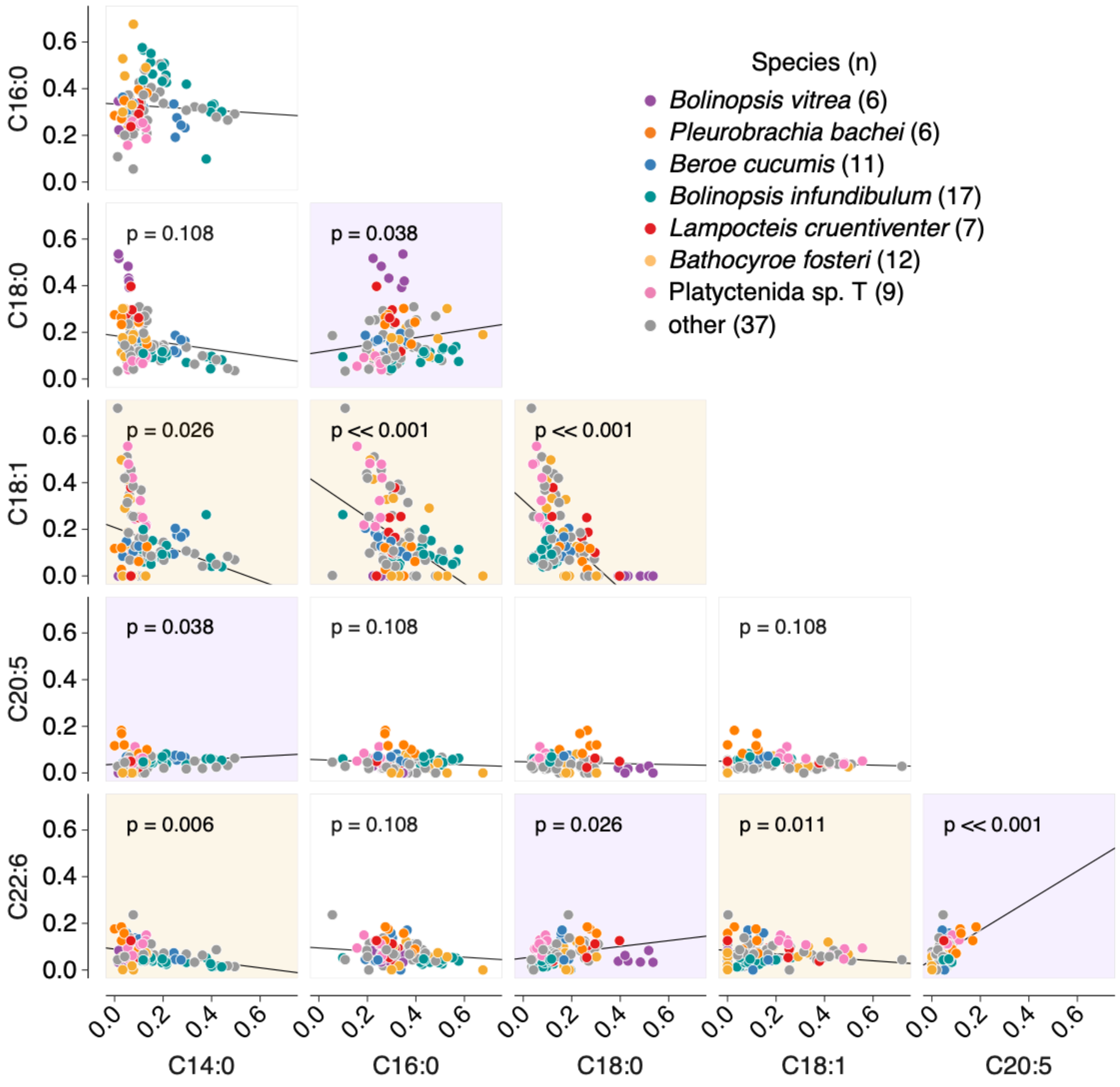

Fig. S4. Intercorrelations of major fatty acid methyl esters suggest metabolic exchanges and functional equivalence

Phylogenetic regressions of molar fraction of each of the predominant six fatty acid methyl esters against each of the others, for assessment of potentially relevant lipid metabolism pathways. All 105 samples are included in every regression, providing further evidence for the importance of the SFA/MUFA balance most likely mediated by ELOVL and SCD1 enzymes. The strong positive relationship between PUFAs C20:5 and C22:6 identifies them as a covarying and perhaps functionally similar unit. Species are color-coded as in figures 2 and 3, with magenta- or orange-shaded plot backgrounds denoting significant positive or negative correlation.

Journal of Experimental Biology • Supplementary information

Table S1. (Excel format): Species summaries of environmental parameters and fatty acid/alcohol compositions
Each row contains data for a single species, with values stated as mean ±SD. Species are ordered by increasing mean depth. A dash in the mean position indicates that a compound was not detected, whereas a dash in the SD position reflects n=1 and consequent lack of a variability statistic. Columns marked with an asterisk were normalized by the total of all compounds detected (even- and odd-chain fatty acids plus fatty alcohols.) All other fatty acid columns were normalized by total even-chain fatty acids, since those compounds are most integral to animal metabolism and comprise the majority fraction of all samples. The raw data and functions used to calculate these summary values are available at http://github.com/octopode/cteno-lipids-2021.

Click here to download Table S1

Table S2. Collection information and assembly statistics for transcriptomes used in provisional phylogeny
Specimens are ordered by species as in Table S1. BW* indicates that a specimen was collected between 0 and 20 m depth using blue-water SCUBA. Corresponding temperature values are means for that depth window measured concurrently by ROV-mounted CTD.

Species	Date collected	Depth (m)	Temperature (°C)	Latitude	Longitude	Reads	Size (Mbp, trimmed)	Contigs	Mean length (bp)
Pleurobrachia bachei	4/19/2019	BW*	12.01	36.4174° N	122.3038° W	53189478	115.02	106955	1075.39
Bolinopsis vitrea	11/5/2018	BW*	27.60	19.7745° N	156.1139° W	58709706	114.07	115335	988.99
Beroe ovata	3/20/2017	BW*	24.46	19.2613° N	156.1495° W	118311284	171.13	200766	852.39
Mertensia ovum	6/1/2016	BW*	5.13	76.0980° N	15.9800° E	684500976	181.79	212890	853.93
Beroe cucumis	3/8/2015	304.00	10.69	24.5010° N	110.0023° W	111028994	158.18	211150	749.12
Beroe forskalii	9/30/2012	BW*	12.61	35.9306° N	122.9327° W	36220776	71.68	62940	1138.81
Hormiphora californensis	6/2/2007	100.00	14.37	26.6660° N	110.1480° W	50066544	119.15	121050	984.29
Bolinopsis infundibulum	4/24/2013	96.57	8.80	36.7037° N	122.0404° W	41132986	78.87	57907	1361.93
Cydippida sp. C	6/11/2004	151.30	8.49	36.7043° N	122.0672° W	48472366	89.51	108667	823.74
Eurhamphaea vexilligera	11/4/2018	BW*	27.40	19.2963° N	157.1638° W	61950638	139.59	133057	1049.13
Lampocteis cruentiventer	9/30/2012	650.74	5.49	35.5640° N	123.7553° W	44174126	82.75	78239	1057.66
Aulacoctena acuminata	11/24/2013	1223.84	3.32	36.6000° N	122.1501° W	57142936	67.16	100954	665.24
Cydippida sp. X	7/13/2013	677.16	4.75	36.1147° N	123.5481° W	34098150	98.67	122974	802.40
Lobata sp. V	12/3/2011	1755.02	2.23	36.0716° N	122.2817° W	44417590	110.65	135999	813.60
Bathyctena chuni	10/1/2012	924.72	4.18	36.2497° N	123.1656° W	34496316	97.77	163110	599.40
Bathocyroe fosteri	4/24/2013	589.36	5.64	36.6981° N	122.0476° W	45792910	89.31	85961	1038.98
Euplokamis dunlapae	12/19/2007	203.87	8.62	36.7813° N	122.0173° W	31539004	55.18	48464	1138.48
Lampea pancerina	7/14/2013	1776.67	2.38	36.3834° N	122.6664° W	51099402	126.13	139158	906.36
Platyctenida sp. T	9/20/2014	3980.88	1.49	35.5001° N	124.0008° W	222387028	182.01	186439	976.25
Ctenophora sp. B	1/23/2007	2966.40	1.64	35.8332° N	122.6671° W	36243502	82.85	85613	967.72

Journal of Experimental Biology • Supplementary information
